# The impact of perceived organizational support and human resources practices on innovative work behavior: does gender matter?

**DOI:** 10.3389/fpsyg.2024.1401916

**Published:** 2024-06-28

**Authors:** Moyassar Al-Taie, Mohammad Nisar Khattak

**Affiliations:** ^1^Department of Management, College of Business Administration, University of Sharjah, Sharjah, United Arab Emirates; ^2^Department of Management, College of Business Administration, Ajman University, Ajman, United Arab Emirates

**Keywords:** perceived organizational support, high commitment human resource practices, innovative work behaviors, gender, UAE

## Abstract

Modern organizations nowadays are striving to survive and thrive within the intense competition, complex environment, and ongoing globalization. Employees’ innovative work behavior has become the primary vehicle for these organizations to achieve this aim. The purpose of this study is to examine the effect of perceived organizational support (POS) and high commitment human resource practices (HCHRPs) on employee innovative work behaviors (IWB) within the moderating role of gender. Data was obtained from 359 academic staff members working in 124 higher education institutions in all emirates of the United Arab Emirates (UAE). Findings revealed that POS and HCHRPs are positively related to employee innovative work behaviors. Moreover, the moderating effect of gender on the direct relationship between POS and employee innovative work behaviors was significant, but not significant on the direct relationship between HRPs and employee innovative work behaviors. Implications of the findings for academics and practitioners were presented, and limitations and future research were discussed.

## 1 Introduction

Globalization and exponential advancement in information and communication technology resulted in making innovation the central vehicle for the modern days’ organizations to reach the success. Consequently, and specifically during the last three decades, innovative work behavior (IWB) has received considerable attention from practitioners, and academics alike (de Jong and Den Hartog, 2010; Peerzadah et al., 2023).

Innovative work behavior is one of the most catching topic of human resource management and organizational behavior research and refers to the ‘intentional introduction of new and useful ideas, as well as set of behaviors needed to develop, launch and implement ideas with an aim to enhance personal and/or business performance’ (de Jong and Den Hartog, 2007, p. 43). In order to better understand the phenomenon of IWB, in the modern days’ organizations, scholars have shown considerable interested in investigating the predictors and outcomes of IWB from both the organizational and individual perspectives (Yuan and Woodman, 2010).

A pioneering study on the determinants of IWB conducted by Mumford et al. (2002) reported a variety of constructs such as strategy, structure, climate practices, group interactions and individual performance capabilities which play a crucial role in disseminating the IWB. More recently, Farrukh et al. (2023) conducted a systematic literature review on IWB spanning the published work on IWB over the last three decades and highlighted a list of constructs including learning orientation, job satisfaction, job involvement, job design, commitment, leadership, psychological empowerment, work engagement, knowledge sharing, person-organization fit and more as antecedents, mediators, moderators and outcomes of IWB. Similarly, another bibliometric analysis of IWB literature published during the last three decades revealed that the most commonly used keywords were performance, transformational leadership and workplace conditions (Peerzadah et al., 2023). Following the same line of inquiry, a systematic review of IWB literature conducted by AlEssa and Durugbo (2022) highlighted somewhat similar set of IWB’s predictors and grouped those into four categories including: (1) learning and leadership which include leadership style, knowledge sharing behavior (2) processes and performances which include job design, performance, HR management practices (3) innovative work characteristics and conditions which include qualities of individuals and the environmental conditions (4) innovative work inhibitors and interdependencies which covers job stressors, injustice, and job insecurity, psychological empowerment and organization development interventions.

Despite the fact that numerous studies endeavored to identify the significant predictors and outcomes of IWB, including the above-mentioned systematic literature review; empirical studies that link perceived organizational support (POS) and high commitment human resources practices (HCHRPs) are still scarce. Recent studies conducted by Ayoub et al. (2023) and Farrukh et al. (2023) highlighted the scarcity of studies on IWB in higher education in general and particularly in the Middle East. Extant literature also revealed the fact that very few studies are conducted on IWB examining the moderating role of gender (AlEssa and Durugbo 2022; Farrukh et al., 2023; Peerzadah et al., 2023). Therefore, this study responds to the above calls and strive to fill the available gap in the IWB literature.

Gregory et al. (2010) suggests that when employees perceive that their organizations care about them, provide them non-judgmental and honest feedback about their work and encourage them to initiate creative ideas, explore new possibilities, solving current and future problems and translate their creative inputs into actions and innovative output increase. Afsar and Badir (2017) pointed out that once the organizations take care of their employees’ needs and well-being, employees feel organizational support which make them more willing and motivated to perform proactive behaviors such as IWB. Similarly, Pohl et al. (2013) asserted that when employees believe that their organization treats them well and values their efforts, they are inclined to devote greater effort toward the organization.

Universities, being the context of this research, consequently, stand to earn from innovative academics in term of updating syllabi to fit with the ever-changing labor market needs and using the innovative teaching and research methods and tools and coping with technological advancement in all of these fields. In addition, today’s universities are becoming an entrepreneurial entity that strive to convert their research outcomes into start-ups in order to maintain a sustainable income, and that cannot occur without having an innovative academic staff.

Thus, the purpose of this study is twofold: first, the impact of POS and HCHRPs on IWB will be tested within the UAE higher education sector. Second, the moderating role of gender on the relationships between POS, HCHRPs and IWB will be explored. In nutshell, the overarching research question of this study is: What is the relationship between POS, high commitment HCHRPs and innovative work behavior, and how is this relationship moderated by gender of the academic members?

Our study complements and contributes to the existing body of research in several ways. First, it contributes to the body of knowledge and filling the gaps that have identified in the IWB literature by investigating the impact of POS and HCHRPs on the IWB. Second, this study intenst to explore the moderating role of gender on the relationship between POS, HCHRPs, and IWB in higher education sector. Third, it will obtain data and share results from the region (i.e., Middle East) and the sector/industry (i.e., Higher Education) that have been identified as the least region and sector in terms of IWB.

The study is structured as follows: First, the relevant IWB literature is reviewed concerning the POS and HCHRPs and gender. A set of hypotheses is drawn from this literature, and then the methodology utilized to test them is discussed. After presenting the findings, the authors then discuss the wider managerial and academic implications of their study, explain the limitations, and present the future research.

## 2 Literature review and hypotheses development

### 2.1 POS and innovative work behaviors

Organizational support theory (Eisenberger et al., 1986) posits that organizational support helps in increasing employees’ well-being, which in turn develops positive perceptions about their employer. Similarly, following the norm of reciprocity, employees who perceive that they receive more support and respect from their organizations are more likely to work well and go an extra-mile for their organizations. This notion is further supported by the socially embedded model of thriving (Spreitzer et al., 2005) which postulates that particular work contexts (i.e., a climate of trust and respect, decision-making discretion) persuade employees’ agentic behaviors which in turn impact on their approach to their jobs. Hence, exhibition of these behaviors, support from the organization and other job resources promote employees’ thriving in the workplace, which plays a pivotal role in their creativity and innovation (Spreitzer et al., 2005; Niessen et al., 2012; Prem et al., 2017; Guan and Frenkel, 2018). Following the norm of reciprocity, when employees get appreciation for their contribution, they perceive that their employer gives them respect and takes care of their well-being (Armeli et al., 1998).

The POS is considered an important job resource from the organization toward its employees, because it helps to fulfill the employees’ need for esteem, affiliation, approval and emotional support (Kurtessis et al., 2017). It has been established in the previous research that higher levels of POS motivate the employees to get involved in voluntary behaviors that are of high benefit for the organizations (Eisenberger et al., 1990; Settoon et al., 1996; Wayne et al., 1997). Similarly, studies have also found that high levels of POS make employees feel obligated to return in the favor of their organization by going an extra mile (Eisenberger and Rhoades, 2001; Rhoades et al., 2001). POS helps in developing the exchange relationship between employees and organizations, which in turn transforms into various tangible and intangible outcomes such as jobs satisfaction, commitment, task performance, and citizenship behaviors (Eisenberger et al., 2002). Further to this argument, one study reported that when organizations show concern for their employees and provide them with honest feedback for their actions, employees are more likely to initiate creative ideas, look for new opportunities, devise solutions for existing and potential problems, and transform their creative input into innovative outcomes (Gregory et al., 2010).

Innovative work behavior is not a one-time discrete activity; rather, it is a continuous process through which employees develop new ideas along with working on their existing ideas and is dependent on developing organizational support (de Jong and Den Hartog, 2007). Therefore, employees are willing to exhibit innovative work behaviors when they perceive strong support from their supervisor and organization, have a high degree of freedom in their work, and the availability of ample resources (Afsar et al., 2016). Fan et al. (2022) found that providing employees with the required innovation resources, and establish comfortable and fair working environment are essential in the chines organizations that seek to promote innovation. It is argued on the basis of the above-described literature that POS helps in convincing employees that they are an integral part of the organization. Therefore, they feel obliged to respond in a similar way to what they receive from their organization. Hence, we postulate the following hypothesis:

*H1*: Employees’ perceived organizational support positively affects the employees’ innovative work behavior.

### 2.2 High commitment human resources practices and innovative work behaviors

In today’s dynamic and ever-changing business environment, innovation is considered to be a key to the success and survival of organizations (Shahzad et al., 2019). Innovation is a broader concept, which encompasses the introduction of new ideas to the existing line of products, processes and numerous other activities (Curado et al., 2018). This helps in improving the employees’ capabilities, which in turn guarantee the success and high marketability of the organization (Seeck and Diehl, 2016). HR practices are considered as a set of practices that include human resource planning, performance management, result-oriented rewards systems, recruitment and selection, learning and development, etc. (Katou and Budhwar, 2015), and these practices help in developing employee developmental behaviors and in turn enhance the organization’s competitive advantage (Jiang et al., 2012).

Human resource is considered as the backbone of any organization. Without having an ample human talent and the associated expertise, it is nearly impossible for an organization to perform well in today’s competitive business context. Therefore, most of the current management literature focuses on the human resource practices (HRPs) to create a fit between the individual and organizational objectives (Ang et al., 2013). High commitment human resources practices (HCHRPs) was used in this study because practicing commitment oriented HRM by the organization will strengthen the employees identification with the organization and encourage them to go an extra mile to achieve its goals (Rubel et al., 2018). Previous research has established a positive relationship between the high-involvement HRPs and contribution of employees toward organizational competitive advantage and innovative capabilities (Huselid, 1995; MacDuffie, 1995; Shahzad et al., 2019; Zhang et al., 2022; Than et al., 2023). Extending this line of enquiry, a recent study found that HRPs (i.e., staffing, training, participatory decision making) positively predict innovative work behaviors with the mediating lens of meaningful work (Singh et al., 2021). These HRPs provide a harmonious environment which helps in improving the employees’ skills, participation in decision making, discretionary efforts, as well as inculcating their operative and learning behaviors (Kang et al., 2012; Prieto and Pérez-Santana, 2014). Therefore, HRPs are the contextual characteristics of an organization that motivate employees to exert higher levels of effort in their work and exhibit innovative work behaviors (Prieto and Pérez-Santana, 2014; Shahzad et al., 2019; Noopur and Dhar, 2020).

Human resource practices truly reflect the organizational strategy, intention and employee/organization mutual relationship (Shipton et al., 2017). Hence, these activities are considered to be the job training and career planning practices that ultimately transform into the success of organizations (Zheng et al., 2006). A study reported that merit-based promotion and innovation-based appraisal and compensation encourage the employees to utilize their innovative and creative skills and knowledge to produce and implement the new ideas (Saunila, 2016). Similarly, participation in decision making, job autonomy, and flexible job design positively influence creativity and innovation in small and medium enterprises (SMEs) (Baggen et al., 2016). Another study confirmed that job autonomy and participation in decision making enhance the employees’ commitment with their organization and their citizenship behaviors (Byaruhanga and Othuma, 2016). The prevalence of all these practices helps in developing the human capital and to enhance the innovative competencies and motivation, which in turn increases the organizational innovation (Raja and Johns, 2010; Razouk, 2011). In light of the above described literature, we postulate the following hypothesis:

*H2*: High commitment human resources practices positively affect the employees’ innovative work behavior.

### 2.3 Moderating role of gender

Gender has been chosen as a moderator in this study because of the scarcity of studies on the predictors of the IWB and the impact of gender differences in the context of Middle East. For decades, most of the studies on IWB are conducted in the western context and the assumption is made that the findings will be true to other context as well. However, studies on cross cultural differences negate this concept and identify the cultural differences which might have impact on the behaviors of the people in that context. This is one of the motives of choosing the gender as moderator in this study to fill this essential gap in literature.

Extent research has established that high levels of POS trigger high involvement of employees in voluntary behaviors, which positively impacts on overall organizational performance (Eisenberger et al., 1990; Settoon et al., 1996; Wayne et al., 1997). According to the reciprocity norm and social exchange theory, in the presence of high level of POS, employees are more likely to respond in a positive way toward the organization and are more likely to go an extra mile for the organization (Eisenberger and Rhoades, 2001; Rhoades et al., 2001). However, studies also reported that men and women respond in different ways to a similar context (Sanchez-Franco et al., 2009; Riquelme and Rios, 2010). The gender role theory suggests that men are more likely to have self-esteem-oriented motivation, while women report more communally-oriented motivation (Good and Sanchez, 2010). Using the framework of gender role theory, studies have reported that women prefer to work in a friendly and supportive environment while men are inclined toward higher levels of independence, autonomy, monetary compensation and responsibilities (Schwalbe and Staples, 1991; Buelens and Van den Broeck, 2007; Gentile et al., 2009). Similarly, meta-analytic findings have also confirmed that women are happier to work with other people and help others than their male counterparts (Konrad et al., 2000). A recent study has reported that shop-level high involvement HRM practices are more strongly and positively related to women’s affective commitment (Shin et al., 2020).

Taking the above described findings of the extant literature on the role of gender at workplace one step further, this study strives to explore the role of gender in expanding the POS and HRM practices in the Middle Eastern context. After reviewing the relevant literature on POS, HRM practices and gender role at workplace, it is highly convincing to say that higher POS and HRM practices will trigger the intent of men toward the innovative work behaviors more than their women counterparts. In the Middle East, the culture is highly collectivist, where individuals feel pride in being part of a family and show a higher degree of cohesiveness with their organizations (House et al., 2004; Gupta et al., 2014). In such a collectivistic culture, initiating and taking part in innovative and entrepreneurial activities are highly challenging for women (Ufuk and Özgen, 2001; Nearchou-Ellinas and Kountouris, 2004). A recent meta-analysis of research on gender discrimination/gender gaps in Science, Technology, Engineering and Mathematics (STEM) in the UAE identified a decreasing trend in gender discrimination; however, the authors argued that the issue still exists (Patterson et al., 2020). Extending this line of inquiry, this study argues that prevalence of higher POS and HRM practices will give more autonomy, independence and support to their employees, however men are more likely to exploit these resources and go an extra mile for their organization by showing a higher inclination toward innovative work behaviors. In contrast, women being fewer in number in most of the organizations and having lesser impact in policy making are less likely to exhibit the innovative behaviors. Hence, we postulate the following hypotheses:

*H3*: Gender moderates the positive impact of POS on employees’ innovative work behaviors such that the relationship will be stronger for male employees than for female employees.

*H4*: Gender moderates the positive impact of HRM practices on employees’ innovative work behaviors such that the relationship will be stronger for male employees than for female employees.

### 2.4 Control variables

**Figure 1:**
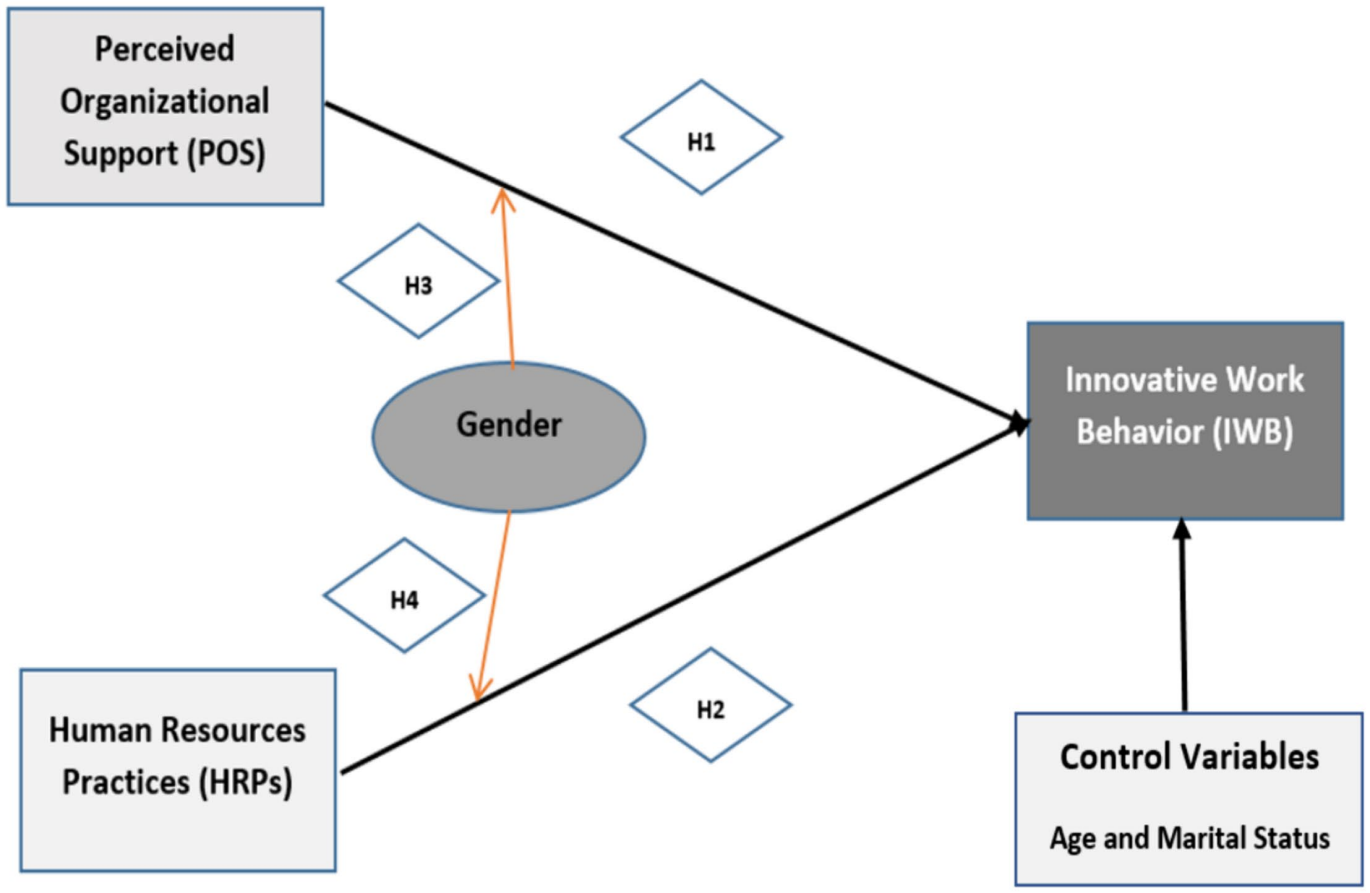
The proposed research model.

To avoid the possibility of having any observed relationships that might impacted by participants’ demographic characteristics, we controlled for employees’ age (in years) and marital status (1 = Married; 2 = Unmarried) because they may account for variation in innovative behavior (Guillén and Kunze, 2019; Yasir and Majid, 2019). Figure 1 below is illustrating the hypothesized relationships in this study.

### 2.5 Context of the study

The UAE is a multicultural country and has a very high influx of expatriates from all around the world in the last few decades (Gaweesh and Haid, 2018). This high influx put enormous pressure on local customs and traditions, however, local people have the capability to maintain their cultural heritage in a successful way (AlMazrouei and Pech, 2015). It is important at this point in time to understand the status of UAE in terms of Hofstede’s cultural dimensions. UAE is high on the index of Power Distance, showing higher hierarchy in culture which is apparent in higher centralization, inherent inequalities, autocratic leadership style and higher obedience from subordinates (Alteneiji, 2015). On the index of individualism/collectivism, UAE is deemed as a high collectivistic society and exhibit higher integration throughout their life time (Hofstede, 2011, p. 92). In addition, on masculinity/femininity index UAE is somewhere in the middle showing higher concerns for the quality of life and achievements and competition (Aljerjawi, 2016). Lastly, UAE is high on avoiding uncertainty showing higher concerns toward traditions and rituals and have higher tendency toward formal and bureaucratic organizational structures (Hofstede, 2011).

## 3 Research methodology

This study’s target population consisted of all local and expat academics working in the UAE’s higher education providers. The unit of analysis was the individual instructors. A quantitative approach, based on the survey method, was used to test the research hypothesis. Over time, the survey methodology has been the most widely used method in innovation research (Ramamoorthy et al., 2005; Montani et al., 2014; Afsar and Rehman, 2015; Afsar and Umrani, 2019; Wynen et al., 2020). An online national and large-scale survey was adopted to collect data related to the variables investigated in this study. The online survey was conducted/administrated by a third party; a well-known, specialist data service and marketing research organization, Dun and Bradstreet using their extensive database that included a list of 18,600 emails for academic staff members working in 124 higher education providers in all emirates of the UAE. This list served as a sampling frame. The UAE academic community can be described as highly divers, therefore, the study population was divided into homogeneous subpopulations (stratum) based on specific characteristics (gender, age, marital status, academic rank, and location), then a convenance sample from each strata were collected. Stratified sampling was adopted to ensure that every characteristic is properly represented in the sample. Table 1 provides a socio-demographic profile of the respondents who participated in this study. With respect to the sample characteristics, the majority of participants were male (67%); 85% of them were married. Additionally, 39% held an assistant professor title, and 98% of them were expats. Finally, 78% of the sample’s respondents were from the UAE major cities of Abu Dhabi, Dubai, and Sharjah.

**Table 1:** A socio-demographic profile of the respondents.

Demographics	Number of people (frequency)	% of total *n*
Gender		
Male	240	67
Female	119	33
Marital Status		
Married	304	85
Single	55	15
Academic Rank		
Professor	67	18
Associate Professor	71	20
Assistants Professor	140	39
Lecture	82	23
Nationality		
Locals	08	02
Expats	351	98
Emirate/City		
Abu Dhabi	76	21
Dubai	94	26
Sharjah	144	40
Ajman	12	03
Al-Fujairah	06	02
Umm Al-Quwain	16	05
Ras Al-Khaimah	11	03

### 3.1 Data collection

Leavy (2017) have recommended using sample size calculators, which are available online, to determine the ideal sample size for a particular study. The targeted sample size was calculated by the sample size calculator provided by Qualtrics (https://www.qualtrics.com/blog/calculating-sample-size/). The ideal sample size was found to be 370 responses, based on a population size of 18,600 and a confidence level of 95%. An invitation email and a URL link were sent to all of the targeted populations during the last quarter of 2019. This was done to obtain the targeted sample size and reach a large enough sample size to use structural equation modeling, as recommended by Tabachnick and Fidell (2007) and Kline (2011). The questionnaire was distributed in English language as it is a widely spoken language in the UAE and because it was the targeted population (academics) main language for teaching/researching in most of the UAE higher education providers. A total of 396 questionnaires were received; however, 37 questionnaires were incomplete. After eliminating the incomplete ones, the final realized sample was 359 usable questionnaires, which was very close to the ideal sample size calculated by Qualtrics. The total number of usable questionnaires also exceeded the rule of thumb of 10 times the largest number of structural paths directed at a particular construct in the structural model (Hair et al., 2017). In our model, there are three paths directed at innovative work behavior; therefore, the minimum sample size should be 30. The sample size of this study is comfortably above this minimum.

### 3.2 Measures

The questionnaire of this study consists of two parts: the first part was including the several questions about the participants demographics (gender, marital status, academic rank, nationality, and location of the higher education institutes they worked for) while the second part included the measurements of the main research variables explained below. The items of these scales were anchored on a six-point Likert scale to avoid neutral responses. Negatively worded items were reverse-scored, so higher scores represent higher levels of the construct being measured. In all such cases, only measures previously reported in the literature as having sound psychometric properties were used to measure the constructs of interest to this study.

*Perceived Organizational Support (POS): The Perceived Organizational Support was measured with an eight-item version of the original 36 items developed by* Eisenberger et al. (1986) *and used by* Eisenberger et al. (1997)*. These eight items were loaded highly on the main factor, which seemed applicable to a wide range of organizations. The following is a sample item: “My organization really cares about my well-being.” Cronbach’s Alpha (α) for this construct was found to be 0.869 in this study.*

*High commitment human resource practices:* *The HCHRPs Practices was measured using the eight items developed by* Gould-Williams and Davies (2005). *The scale was based on those HRM practices identified by advocates of the ‘high commitment’ approach* (Wood and Albanese, 1995; Guest, 1997; Guest and Conway, 1997; Pfeffer and Jeffrey, 1998; Marchington and Grugulis, 2000). *The following is a sample item: “I am provided with sufficient opportunities for training or development.” Cronbach’s Alpha (α) for this construct was found to be 0.834 in this study*.

*Innovative Work Behavior (IWB): Innovative work behavior was measured using the nine-item scale developed by* Janssen (2000). *The nine items evaluate the respondents IWB over three stages namely idea generation, idea promotion and idea realization. Following is a sample item: “with what frequency do you engage in creating new ideas for difficult issues?.” Cronbach’s Alpha (α) for this construct was found to be 0.931 in this study*.

### 3.3 Data analysis and hypotheses testing

We prepared the survey data for data analysis by correcting errors, checking and treating outliers, checking for normal distribution, and multicollinearity based on the guidelines provided by Tabachnick and Fidell (2007). Missing values were very few and it was handled using mean substitution option. Armstrong and Overton (1977) recommended procedures to be used to check for possible non-responses. By conducting the Mann–Whitney *U* test, we found no significant differences between the first third and the last third of the respondents’ data, so we concluded that non-response bias did not appear to be an issue in this study. We also checked for common method bias (CMB) in the measurement model for this study’s principal constructs. Harman’s single factor test was used for exploratory factor analysis in IBM SPSS version 24 (Podsakoff et al., 2003). Researchers have argued that if there is a detrimental level of common method bias, ‘(a) a single factor will emerge from the exploratory factor analysis (unrotated) or (b) one general factor will account for the majority of the covariance among the measures’ (Podsakoff et al., 2003, p. 889), as four factors emerged from an exploratory factor analysis (unrotated) to explain 76.3% of the variance. The first factor explained only 31.51% of the variance; thus, we can infer that common methods bias was not an issue in this study. A common method bias (CMB) for the constructs included in this study were also checked by conducting full collinearity assessment based on the instruction provided by Kock (2015, p. 7) and using SmartPLS. The outcomes of this test revealed that all variance inflation factors (VIFs) for this study latent variables were lower than the threshold of 3.3; thus, we could infer that common methods bias was not an issue in this study.

Once both of the control variables in this study are categorical in nature. Therefore, an ANOVA analysis were conducted using the categorical control variables as independent variables (i.e., marital status, and age) and IWB as a dependent variable. None of the categorical control variables were significant and were excluded from further analysis.

Data analysis was run using Partial Least Square Structural Equation Modelling (PLS-SEM). SmartPLS 3.2.9 software (Ringle et al., 2015) was used to analyze the data. PLS-SEM was selected because it is suitable for testing theoretical frameworks from a prediction perspective, a distributional-free technique, and can reach high levels of statistical power even with a limited sample size like ours (Hair et al., 2019). The results of the PLS analysis were used to test the research hypotheses. Guidelines provided by Chin (2010) and Hair et al. (2016) were followed to analyze the data over the two stages: testing the measurement model and then testing the structural model.

### 3.4 Measurement model

Following the common criteria that Hair et al. (2016) have suggested for the reflective measurement model, we examined internal consistency (composite reliability), indicator reliability, convergent validity (average variance extracted), and discriminant validity. Convergent validity was assessed through factor loadings, composite reliability (CR), and Average Variance Extracted (AVE). Table 2 shows that all item loadings exceeded the recommended value of 0.6 (Chin, 1998) except for four items. A decision was made to eliminate these four items from further analysis. The composite reliability values for all constructs exceeded the recommended value of 0.7, indicating a high level of reliability. The Average Variance Extracted (AVE) for all constructs also exceeded the threshold of 0.50, which suggests that each adequate convergent validity explains at least 50 percent of the variance of its items.

**Table 2:** Construct reliability and validity.

Factors	Item loading	Cronbach’s alpha	CR	AVE
IWB		0.931	0.932	0.644
IWB1	0.752			
IWB2	0.790			
IWB3	0.754			
IWB4	0.853			
IWB5	0.781			
IWB6	0.820			
IWB7	0.854			
IWB8	0.856			
IWB9	0.818			
HRPs		0.834	0.878	0.524
HEP1	0.812			
HRP2	0.817			
HRP3	0.143			
HRP4	0.584			
HRP5	0.725			
HRP6	0.831			
HRP7	0.683			
HRP8	0.767			
POS		0.869	0.899	0.549
POS1	0.873			
POS2	0.825			
POS3	0.712			
POS4	0.824			
POS5	0.817			
POS6	0.234			
POS7	0.493			
POS8	0.836			

Shaded items representing weak items which are eliminated from further analysis. AVE, average variance extracted; CR, composite reliability.

Next, we assessed the discriminant validity, aiming to determine the extent to which a construct was empirically distinct from other constructs in the structural model. The correlation matrix exhibited in Table 3 shows that the square root of the AVE (diagonal values) of each construct was larger than its corresponding correlation coefficients, confirming an adequate discriminant validity (Fornell and Larcker, 1981). Henseler et al. (2015) have argued that the Fornell and Larcker criterion does not perform well, particularly when the items’ loadings on a construct differ only slightly; therefore, they proposed the Heterotrait-monotrait Ratio (HTMT) of the correlation as a better replacement. Once our items’ loadings on their respective constructs differed slightly, the discriminant validity was tested using the new technique. The results of the Heterotrait-monotrait Ratio (HTMT) test, as shown in Table 4, confirm an adequate discriminant validity as all values in the table are below the threshold value of 0.85 (Kline, 2011).

**Table 3:** Inter-construct correlation matrix.

Construct*	IWB	POS	HRPs
IWB	0.815**		
POS	0.593	0.766	
HRPs	0.570	0.682	0.72

*Six-point Likert scale, **Square root of AVE in diagonal.

**Table 4:** Heterotrait–monotrait ratio (HTMT).

Construct	IWB	POS	HRPs
IWB			
POS	0.632		
HRPs	0.616	0.742	

### 3.5 Structural model

Having established confidence in the measurement model, the structure model needs to be tested to examine the hypothesized relationships depicted in the research model. Hair et al. (2016) have suggested examining five indicators to test the structural model: coefficients of determination (*R*^2^); predictive relevance (*Q*^2^); Size and significance of path coefficients (Beta-β); *f*^2^ effect sizes; *q*^2^ effect sizes. First, we examined the direct relationship between the variables.

**Figure 2:**
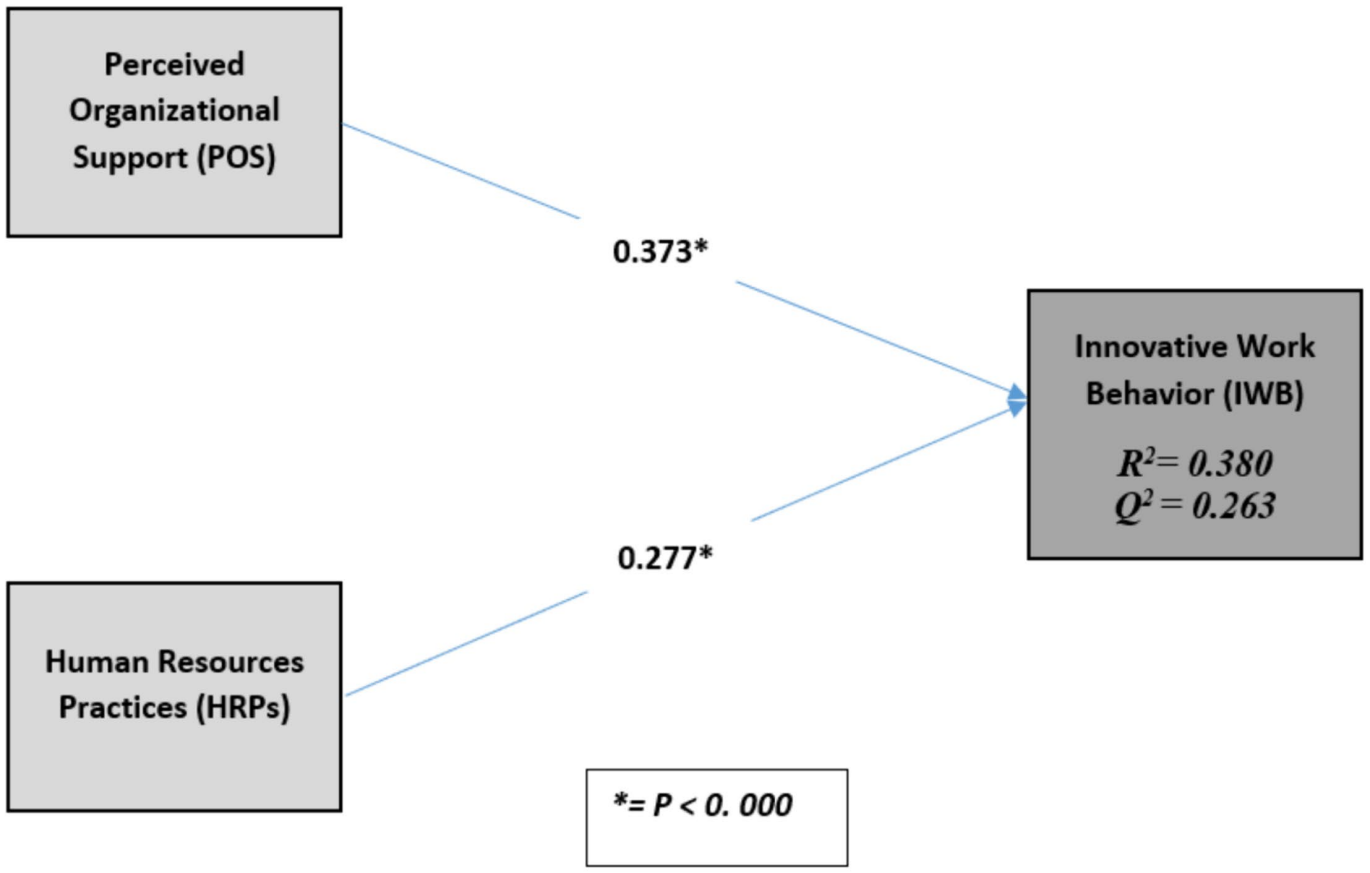
PLS structural model results.

Results indicated that both HRPs and POS significantly correlated with employee innovative behavior (*β* = 0.277; *p* < 0. 000; *B* = 0.373; *p* < 0. 000); thus, we accept research hypotheses one (H1) and two (H2). Table 5 and Figure 2 summarizes these results. Moreover, HRPs and POS explain 38% of the variance in innovative work behavior (*R*^2^ = 0.380). The *R*^2^ value of 0.380 is higher than the 0.26 value that Cohen (1988) suggested would indicate a substantial predictive power. To test the effect size, Cohen’s (1988) guidelines were used, which are 0.02 for a small effect, 0.15 for a medium effect, and 0.35 for a large effect. Table 5 shows that the two hypothesized relationships had a small effect size. Along with *R*^2^ and *f*^2^, the PLS path model’s predictive accuracy was checked by calculating the *Q*^2^ using blindfolding procedures. Results show the endogenous variable (IWB) in this study had an acceptable predictive relevance as their *Q*^2^, as shown in Table 5, which was greater than zero.

**Table 5:** Structural estimates- research hypotheses testing.

Hi	Path	Beta (β)	*T*-Statistic	Decision	*f* ^2^	*R* ^2^	*Q* ^2^
H1	HRPs → IWB	0.277	3.652**	**Supported**	0.046	**0.380**	**0.263**
H2	POS → IWB	0.373	5.221**	**Supported**	0.083		

Critical *t*-values. **2.58 (*p* < 0.01). Bold values means represented the significant relationships only that supported our hypotheses.

To test gender differences, a multi-group analysis (PLS-MGA) was conducted after we created two groups: Male (1) and Female (2). To check whether the differences between the two groups were significant, we checked the parametric test results obtained from the PLS/MGA results report, as depicted in Table 6.

**Table 6:** Bootstrapping results for two groups’ differences.

Hi	Path	Beta (β) (male – female)	*T*-Statistics	Result
H3	HRPs → IWB	−0.252	1.702^n.s^	Not supported
H4	POS → IWB	**0.341**	2.351*	**Supported**

Critical *t*-values. *1.96 (*p* < 0.005); n.s, not significant. Bold values represented the significant relationships only that supported our hypotheses.

As can be seen in the table above, it was found that the differences between the two sexes was significant only in the perceived support they received from their organizations. The results indicated that the female employees seemed to receive significantly less support from their organizations than their male co-workers. This finding provides support for research hypothesis three (H3) but not for research hypothesis four (H4).

## 4 Discussion

This study has explored the impact of POS and HRPs on employees’ innovative work behaviors. In modern-day practices, managers and policy makers are seriously concerned about organizational support and HR practices in order to increase the desired employees’ innovative work behaviors. It is evident from the results of this study that high organizational support is the strongest predictor of employees’ innovative work behaviors. This implies that employees, who are given ample support by their organizations, are ready to bring innovative ideas to the table, which might play a pivotal role in bringing competitive advantage to organizations. More specifically, when organizations take care of their employees’ well-being and needs, they feel a higher degree of empowerment and support from their organization, which might induce them to exert higher level of effort and might keep them motivated to continue to exhibit innovative and proactive behaviors.

### 4.1 Theoretical implications

Findings of this study, related to the positive relationship between perceived organizational support and employees’ innovative work behaviors, are consistent with previous studies (i.e., King-Kauanui et al., 2005; Afsar and Badir, 2016; Nazir et al., 2019). Moreover, results of this study also support the notion of innovative work behaviors as a continuing process (Spreitzer, 1995), which always requires organizational support to be effective. Therefore, it is evident that POS provides a conducive environment to employees in which innovative work behaviors might flourish.

The second important objective of this study was to evaluate the impact of HRPs on employees’ innovative work behaviors. Findings revealed that HRPs positively predicted employees’ innovative work behaviors. In other words, HRPs comprising teamwork, employee involvement, empowerment, offer of fair rewards and job security, played a key role in facilitating innovative behaviors in organizations. Exhibition of these practices by organizations enhanced the sense of ‘employees’ oneness with organizations’, which in turn motivated them with regards to innovative behaviors. Results of this study corroborate findings of previous studies which found that intrinsic motivation, rewards and incentive systems, evaluation and job design, and high involvement HR practices significantly predict employees’ innovative behaviors (Amabile et al., 1996; Oldham and Cummings, 1996; Prieto and Pérez-Santana, 2014).

Lastly, we also explored the moderating impact of gender on the positively relationship between POS, HRPs and employee innovative work behaviors. It is evident from labor economics and management literature that the gender gap is an important and well-explored topic of research. However, there are very few studies on this topic in the Gulf region [Gulf Cooperation Council (GCC)] and the UAE (Patterson et al., 2020). Patterson et al. (2020) conducted a meta-analysis on the patterns in a detailed literature review on gender roles in MENA and GCC countries, and they found that gender discrimination is a decreasing trend; however, the issue still exists in the region. Findings of this study support this notion and show that males are more prone toward innovative work behaviors than their female counterparts. This is logical because males are dominant in most of organizations in this region and therefore get more support from their organizations and more benefits as such. Such an environment provides a more suitable platform for male rather than female employees to utilize their innovative skills to its full potential and exhibit innovative work behaviors.

### 4.2 Practical implications

This study has a number of valuable implications for managers/practitioners and policy makers. First, managers at the top-level hierarchy of organizations (i.e., educational institutions) are encouraged to develop and maintain a supportive work environment for employees so that they have access to the valuable resources in their respective organizations, which is highly likely to lead them toward innovative work behaviors. Eisenberger et al. (1986) asserted that three general forms of perceived favorable treatment received from the organization (i.e., fairness, supervisor support, and organizational rewards and job conditions) represent the antecedents of POS. This emphasizes that treating the employees fairly, encouraging the academic department chairs to provide continuous support for them, along with a fair and motivational organizational reward system, as well as improving the quality of working life, are likely to contribute to maintaining high levels of innovative work behaviors. Such a supportive environment develops a sense of oneness in employees with their organizations and, as a result, they are more likely to go the extra mile for the organizational benefit. Therefore, managers and practitioners setting at the responsible positions in organizations are encouraged to provide access to employees to the valuable resources of the organization and give them higher degree of autonomy and participation in decision making. All these will enhance their morale and motivation and lead them toward bring in new ideas into their organization. Second, managers are also encouraged to exhibit HRPs such as continuous learning, interdisciplinary training, promote collaboration and teamwork, Facilitate networking opportunities with industry experts and other educational institutions, employee involvement and empowerment, redesign the reward system to foster innovation performance. Following the norm of reciprocity and social exchange theory (Blau, 1964), these motivated employees will be more likely to display higher levels of effort in their work. Hence, it should be one of the primary responsibilities of managers to develop and environment where employees have a higher trust on each other and are ready to go above and beyond the call of their duties for the organizational benefits. Lastly, and as far as the gender differences is concerned, top management and human resource managers should highlight the significance of POS to both males and female and ensure gender equality in organizations to get the maximum benefits from the their human capital. It is recommended to ensure the equity and equality in organizations at all levels to foster innovative work behaviors.

### 4.3 Limitations and future research directions

Despite the strengths of this study, there are also a number of limitations associated with it which must be kept in mind before generalizing the results. First, cross-sectional data were collected to test the hypotheses of this study rather than time-lagged data, and therefore, the causal relationships were tested in a single study at one point in time, rather than at different points in time. The common method bias test was conducted using two different methods to avoid the effect of this design issue. Although the sample size of this study was acceptable (359), a bigger sample would have allowed us to run a more powerful analysis. Future researchers are encouraged to collect longitudinal data to address this issue. Another potential shortcoming in this study was conducting it in a single context (higher education sector), which might reduce the generalizability of its results to other contexts. The above limitations, along with the gaps identified in the comprehensive literature review, will open the door for future research to address these gaps. Future studies, set in the context of the manufacturing/services sectors, may provide new insights into the innovative work behaviors of employees.

## 5 Conclusion

This study has investigated the moderating effects of gender on the positive relationship between POS, HRPs and employees’ innovative work behaviors. By showing that males are more inclined toward innovative work behaviors in a highly supportive organizational environment, this study has proposed that a supportive environment can be used to encourage the involvement of males in innovative work behaviors.

## Data availability statement

The raw data supporting the conclusions of this article will be made available by the authors, without undue reservation.

## Ethics statement

Ethical review and approval was not required for the study on human participants in accordance with the local legislation and institutional requirements. Written informed consent from the [patients/participants OR patients/participants legal guardian/next of kin] was not required to participate in this study in accordance with the national legislation and the institutional requirements.

## Author contributions

MA-T: Conceptualization, Data curation, Formal analysis, Funding acquisition, Investigation, Methodology, Project administration, Software, Supervision, Validation, Visualization, Writing – review & editing. MK: Funding acquisition, Resources, Writing – original draft, Writing – review & editing.
